# An innovative 4D printing approach for fabrication of anisotropic collagen scaffolds

**DOI:** 10.1088/1758-5090/ad7f8f

**Published:** 2024-10-24

**Authors:** Nashaita Y Patrawalla, Karly Liebendorfer, Vipuil Kishore

**Affiliations:** 1 Department of Biomedical Engineering and Sciences, Florida Institute of Technology, Melbourne, FL 32901, United States of America; 2 Department of Chemistry and Chemical Engineering, Florida Institute of Technology, Melbourne, FL 32901, United States of America

**Keywords:** 3D printing, 4D printing, collagen, extrusion, magnetic alignment, ligaments, tissue engineering

## Abstract

Collagen anisotropy is known to provide the essential topographical cues to guide tissue-specific cell function. Recent work has shown that extrusion-based printing using collagenous inks yield 3D scaffolds with high geometric precision and print fidelity. However, these scaffolds lack collagen anisotropy. In this study, extrusion-based 3D printing was combined with a magnetic alignment approach in an innovative 4D printing scheme to generate 3D collagen scaffolds with high degree of collagen anisotropy. Specifically, the 4D printing process parameters—collagen (Col):xanthan gum (XG) ratio (Col:XG; 1:1, 4:1, 9:1 v/v), streptavidin-coated magnetic particle concentration (SMP; 0, 0.2, 0.4 mg ml^−1^), and print flow speed (2, 3 mm s^−1^)—were modulated and the effects of these parameters on rheological properties, print fidelity, and collagen alignment were assessed. Further, the effects of collagen anisotropy on human mesenchymal stem cell (hMSC) morphology, orientation, metabolic activity, and ligamentous differentiation were investigated. Results showed that increasing the XG composition (Col:XG 1:1) enhanced ink viscosity and yielded scaffolds with good print fidelity but poor collagen alignment. On the other hand, use of inks with lower XG composition (Col:XG 4:1 and 9:1) together with 0.4 mg ml^−1^ SMP concentration yielded scaffolds with high degree of collagen alignment albeit with suboptimal print fidelity. Modulating the print flow speed conditions (2 mm s^−1^) with 4:1 Col:XG inks and 0.4 mg ml^−1^ SMP resulted in improved print fidelity of the collagen scaffolds while retaining high level of collagen anisotropy. Cell studies revealed hMSCs orient uniformly on aligned collagen scaffolds. More importantly, collagen anisotropy was found to trigger tendon or ligament-like differentiation of hMSCs. Together, these results suggest that 4D printing is a viable strategy to generate anisotropic collagen scaffolds with significant potential for use in tendon and ligament tissue engineering applications.

## 1. Introduction

Accurate biomimicry of native tissue is the cornerstone for generating scaffolds with the essential physicochemical cues (e.g. composition, anisotropy) to direct tissue-specific cell fate [1, 2]. For tendon and ligament tissue engineering applications, use of collagen as a biomaterial is a popular option for multiple reasons that include ease of processing, availability of cell adhesion sites, and the fact that the compositional aspects of collagenous scaffolds are akin to the native tissue [3, 4]. Apart from composition, the microarchitecture is of paramount importance to mimicking the anisotropic properties as well as the 3D complexity of the native tissue [5, 6]. Matrix anisotropy in collagen scaffolds has been shown to stimulate tissue-specific cell response as evidenced by preferential cellular alignment, increased proliferation and differentiation, and formation of *de novo* cell-made oriented matrix [6, 7]. While collagen fiber alignment can be triggered using mechanical, electrical, or magnetic stimulus, these methods are associated with drawbacks which include (1) laborious post-processing steps on the 2D fibrous or sheet-like materials to deliver an implantable 3D scaffold [8], (2) some of the collagen alignment methods (e.g. electrospinning) may denature the native protein structure due to limitations of the fabrication process and the use of harsh chemical solvents [9], and (3) limited control over generating customizable scaffolds which can be achieved by 3D printing techniques. Therefore, there is a need for innovative and advanced biomanufacturing techniques to circumvent the challenges associated with existing methods and develop 3D collagenous scaffolds with tissue-mimicking anisotropic properties for use in tendon and ligament tissue engineering applications.

The evolution of 3D printing technologies has revolutionized the field of tissue engineering and regenerative medicine by enabling layer-by-layer fabrication of patient-specific customizable scaffolds with complex geometry [10, 11]. In this realm, extrusion-based bioprinting is a widespread additive manufacturing technique that entails the use of shear-thinning hydrogel-based bioinks for the generation of 3D scaffolds that match the contour and complexity of the native tissue. For example, Klarmann *et al*, have recently shown that by using extrusion-based printing and collagen ink, it is feasible to print scaffolds that mimic the native architecture of human meniscus tissue [12]. However, the resultant scaffolds lack collagen anisotropy, a critical biophysical factor to drive material-directed cell response and remodeling [13, 14]. Application of 4D printing methodologies to induce time-dependent *in situ* scaffold remodeling in response to external stimulus such as temperature, light, and electrical and magnetic fields have been shown to yield shapeshifting or ‘smart’ hydrogel-based scaffolds [15–18]. Leveraging the 4D printing approach can facilitate remodeling of the collagen construct during the printing process to deliver 3D complex scaffolds with the desired anisotropic properties for use in soft tissue applications such as tendons and ligaments [19, 20].

The goal of the current study was to combine extrusion-based 3D printing and magnetic alignment approach in an innovative 4D printing scheme to guide real-time matrix remodeling and deliver 3D anisotropic collagen scaffolds. Specifically, methacrylated collagen (Col) ink was mixed with xanthan gum (XG) and streptavidin-coated magnetic particles (SMPs) and exposed to a constant magnetic field (0–0.2 Tesla) during the printing process. The process parameters (i.e. Col:XG ratio, SMP concentration, print flow speed) were systematically modulated and their impact on rheological properties, print fidelity, and degree of collagen anisotropy was investigated. In addition, the effects of collagen anisotropy on human mesenchymal stem cell (hMSC) morphology, metabolic activity, and tenogenic differentiation were assessed. Outcomes of this work have the potential to advance the field of biomanufacturing by delivering a new 4D printing technology to enable the generation of biomimetic collagen scaffolds with the essential biophysical and biochemical cues for use in tissue engineering applications.

## 2. Materials and methods

### 2.1. Materials

Methacrylated type I collagen (Col) was purchased from Advanced Biomatrix (San Diego, CA). XG from Xanthomonas campestris was acquired from Sigma Aldrich (St. Louis, MO). 2,2′-Azobis[2-methyl-N-(2-hydroxyethyl)propionamide] (VA-086) photoinitiator was purchased from Waco Pure Chemical Corporation (Tokyo, Japan). Streptavidin MagneSphere Paramagnetic Particles (SMP; 1 mg ml^−1^) were purchased from Promega (Madison, WI). Human MSCs (PT-2501) were purchased from Lonza (Allendale, NJ). All other chemicals and reagents were purchased from Fisher Scientific (Waltham, MA) unless stated otherwise.

### 2.2. Effects of Col:XG composition and SMP concentration on rheological properties

Rheological properties of different ink compositions were measured using a Discovery Series HR30 Rheometer (TA Instruments, New Castle, DE, USA) (*N* = 5/group). Briefly, acid soluble Col (6 mg ml^−1^ in 20 mM acetic acid) was neutralized and mixed with XG (10 mg ml^−1^ in water) in varying ratios of Col:XG (1:1, 4:1, or 9:1 v/v) in a microcentrifuge tube, to which a cytocompatible photoinitiator VA-086 (1% w/v of collagen solution) and SMP (0, 0.2, or 0.4 mg per ml of collagen solution) were added. Approximately 350 *µ*l of each of the inks was loaded on to a 20 mm sandblasted steel platform and oscillation strain sweeps were performed between 0.001% and 200% at a constant frequency of 1 Hz to determine the yield stress (YS). YS is defined as the point of crossover of the storage modulus (*G*′) and loss modulus (*G*″) or the point at which *G*″ becomes greater than *G*′ and the material loses its viscoelastic properties. *YS* was calculated using equation (1), where $\tau $ denotes the oscillation torque at which the crossover takes place and *A* is the area of the steel platform on to which the inks are loaded.

\begin{align*}{\text{YS}} = \tau /{\left( {\pi *A} \right)^2}\end{align*}

To assess the shear thinning properties, inks were loaded on to the steel platform and the apparent viscosity was measured by varying the shear rate from 0.001 to 200 s^−1^. A logarithmic plot of apparent viscosity vs. shear rate was generated to determine the power law index (*n*) given by the slope of the curve, and consistency (*K*) of the inks given by the *y*-intercept of the curve at a shear rate of 0 s^−1^. The viscosity (*η*) was calculated at a shear rate $\left( \gamma \right)$ of 10 s^−1^ using equation (2) [21],

\begin{align*}\eta = K{\gamma ^{n - 1}}\end{align*}

### 2.3. Preparation of 4D printed collagen scaffolds

Acid soluble Col (6 mg ml^−1^) was neutralized and mixed with XG (10 mg ml^−1^) in varying ratios of Col:XG (1:1, 4:1, or 9:1 v/v) in a 5 ml syringe. Cytocompatible photoinitiator VA-086 (1% w/v of collagen solution) and SMP (0, 0.2, or 0.4 mg per ml of collagen) were added to mixture and mixed thoroughly and loaded on to an extrusion-based 3D bioprinter (Regemat 3D, Bio V1; Granada, Spain) to print 3D scaffolds from a self-generated STL file according to the printing parameters outlined in table 1. A cylindrical neodymium (N50) magnet (diameter = 3 in, thickness = ¼ in) was attached to the base plate of the printing platform to generate a magnetic field of approximately 0.2 Tesla during the printing process. The printed scaffolds were allowed to incubate on the printer with the magnet for 30 min post printing to allow for the magnetic alignment process. Following this, the scaffolds were incubated at 37 °C for 45 min to promote fibrillogenesis and complete gelation. Lastly, the scaffolds were exposed to UV light (365 nm, 17 mW cm^−2^) for 1 min to photochemically crosslink the printed collagen scaffolds. Scaffolds printed with SMP and without the presence of the magnetic field were used as control.

**Table 1.** Print parameters to fabricate magnetically aligned collagen scaffolds.

Parameter	Dimension	Description
Layer height	0.25 mm	A blunt syringe tip for the printhead
Number of layers	4	Total 1 mm layer height
Needle tip gauge	25 G	Nozzle size to allow for printing with SMP
Print shape	Cuboid	3D construct
Print dimensions (*l* × *w* × *h*)	7 × 7 × 1 mm	Sizeable construct with minimized collagen use
Infill angle	90°	Angle of extrusion of the ink
Flow speed	2 or 3 mm s^−1^	Extrusion speed for print fidelity comparison optimal fidelity of scaffolds

### 2.4. Assessment of print fidelity of 4D printed collagen scaffolds

To assess the effects of different Col:XG ink ratio and SMP concentration on the printability of collagen scaffolds, print fidelity of 4D printed scaffolds was measured. Collagen scaffolds were printed using a flow speed of 3 mm s^−1^ in the presence of the magnetic field. High resolution images of the scaffolds were taken from a fixed distance immediately after printing and the transverse sectional area (TSA) of the printed scaffolds were measured by tracing the 2D surface area of the printed scaffold using ImageJ (NIH, Bethesda, MD). Print fidelity was determined by comparing the TSA of the printed scaffold (*N* = 6/group) to the original STL file generated model (*N* = 1).

### 2.5. Assessment of collagen fiber alignment using polarized light microscopy (PLM)

The impact of Col:XG composition and SMP concentration on collagen fiber alignment was assessed via PLM (*N* = 6/group). 4D printed scaffolds were placed on a glass slide immediately post fabrication and observed under a light microscope incorporated with a polarized lens adaptor (Zeiss AX10 Observer A1). Fiber anisotropy was detected by exploiting the birefringent properties of collagen which cause the scaffolds to show a uniform bright yellow color when the plane fiber orientation is parallel to the plane of polarized light.

### 2.6. Assessment of collagen fiber alignment using scanning electron microscopy (SEM) and quantification of fiber alignment using fast Fourier transform (FFT)

Collagen fiber alignment in 4D printed scaffolds was confirmed using SEM (*N* = 6/group). Briefly, the printed scaffolds were exposed to serial dehydration in different concentrations of ethanol (20%, 50%, 75%, 90%, and 100%) for 15 min at each concentration and then left for 1 h in 100% ethanol. The scaffolds were then placed in a critical point drying system (CPD300, Leica Microsystems) and exposed to 12 cycles of CO_2_ drying, sputter coated with gold for 1 min (Denton Vacuum, LLC) and imaged at 3000× magnification under an SEM (JEOL JSM-6380LV).

The degree of collagen fiber alignment was quantified from SEM images using a total of 15 images per group (*N* = 6/group) and processed using CytoSpectra, an FFT spectral analysis software, and CurveAlign, a curvelet transform analysis software. The distribution of orientation angles of the individual collagen fibers was obtained as 360° plots using CytoSpectra.

### 2.7. Modulation of print flow speed to generate scaffolds to improve print fidelity

The print fidelity of 4D printed scaffolds that yielded the highest degree of collagen alignment was suboptimal when compared to the initial generated STL model. Therefore, to improve the fidelity of the printed scaffolds while preserving the collagen fiber alignment, the print flow speed was modulated by controlling the flow rate of the ink through the nozzle (i.e. 2 mm s^−1^). The thickness of the printed scaffolds was measured using a confocal microscope by scanning through the layers in the *z*-direction. High quality images of the printed scaffolds were obtained and two edge lengths in the *x*-direction and two edge lengths in the *y*-direction were measured, each direction was averaged separately, and this value was multiplied with the thickness to calculate the CSA in the *x*–*z* and *y*–*z* planes. In addition, the TSAs of the printed scaffolds were measured by tracing the 2D surface area of the printed scaffold using ImageJ. The volume of the printed scaffold was given by multiplying the thickness with the TSA. The thickness, TSA, CSA in both planes, and volume of the printed scaffold were compared to the respective values from the generated STL model to determine the overall scaffold print fidelity. Scaffolds fabricated using the modulated print flow speed were analyzed using PLM and SEM to ensure that the new printing parameters did not impact collagen alignment.

### 2.8. Cell culture

hMSCs were cultured in 75 cm^2^ flasks and maintained in mesenchymal stem cell growth medium composed of 10% fetal bovine serum (FBS), 2% L-Glutamine, and 0.1% Gentamycin Sulfate in 5% CO_2_ at 37 °C (PT 3001; Lonza, Allendale, NJ). Passage five cells were used for all the experiments. Cell studies were performed on two groups: (1) collagen scaffolds printed using 4:1 Col:XG composition and 0.4 mg ml^−1^ SMP in the presence of the magnetic field (aligned), and (2) the same scaffolds printed without the magnetic field (unaligned). Prior to culture, collagen scaffolds were submerged in 70% ethanol for 10 min for sterilization and washed three times with sterile 1× phosphate buffered saline (PBS). Following sterilization, collagen scaffolds were placed individually in a 24-well plate coated with poly(hydroxyethyl methacrylate) to prevent cell adhesion onto the well surface. A suspension of hMSCs was prepared in low-glucose Dulbecco’s Minimum Essential Media (DMEM) composed of 10% FBS and 1% penicillin-streptomycin. The cell suspension was pipetted onto the scaffolds at a seeding density of 5000 cells cm^−2^ (based on the area of the well) and cultured for 10 d. The culture medium was first replaced at 6 h to remove the unattached cells, and then every 3 d for the duration of the culture.

### 2.9. Assessment of cell morphology on 4D printed collagen scaffolds

The effect of collagen fiber orientation on cell morphology and alignment was evaluated using fluorescence microscopy (*N* = 8/group/timepoint). Briefly, at periodic intervals (day 3, 7, and 10), culture media was aspirated from the 24-well plate and the scaffolds were washed in 1× PBS. Following this, the cells were fixed by adding 500 *µ*l of 3.7% formaldehyde prepared in 1× PBS for 15 min. The fixative was removed, and the cells were permeabilized in 500 *µ*l of 0.1% Triton-X in 1× PBS solution for 15 min. The permeabilization buffer was then removed and 500 *µ*l of Alexafluor-488 Phalloidin stain solution (Invitrogen, CA) was added. After 30 min, the stain solution was removed, and the scaffolds were washed in 1× PBS and imaged using a fluorescence microscope (Keyence, Osaka, Japan).

Cell orientation was quantified on 5 images per construct (20 images/group) using CytoSpectra to run an FFT-based analysis and deliver 360° angular distribution plots composed of the individual orientation angles of the collagen fibers. Additionally, the CTFire program within CurveAlign was run to deliver values of circular variance between 0 and 1 where 0 indicated highest degree of anisotropy and 1 indicated no preferred direction of cellular orientation.

### 2.10. Impact of collagen fiber alignment on cell metabolic activity

AlamarBlue (AB) assay was performed to analyze the effect of collagen alignment on hMSC metabolic activity (*N* = 12/group/timepoint). AB working solution was prepared by combining AB reagent with culture media in a 1:9 ratio. On days 1, 4, 7, and 10, the culture media was removed and 500 *µ*l of the AB working solution was added to each well and incubated for duration of 2 h at 37 °C. Following this, 100 *µ*l of the solution from each well was added to a bottom clear 96-well plate in triplicates and the fluorescence was measured at an excitation wavelength of 555 nm and an emission wavelength of 595 nm (SpectraMax M2e plate reader, Molecular Devices).

### 2.11. Evaluation of cell phenotype using immunofluorescence

Immunofluorescence staining was performed to assess the expression of scleraxis (SCXA), an early marker for tenogenic/ligamentous differentiation of MSCs [22, 23]. At periodic intervals (days 3, 7, and 10; *N* = 4/group/timepoint), the scaffolds were fixed in 4% paraformaldehyde in 1× PBS for 15 min. Following this, the scaffolds were incubated in permeabilization buffer, prepared with 0.1% Triton-X in 1× PBS for 15 min and then submerged in blocking buffer prepared in the permeabilization buffer with 10% goat serum for 30 min. The scaffolds were then incubated with primary antibody—rabbit anti-human SCXA (Abcam, San Francisco, CA; 1:100 dilution)—in 1× PBS with 10% goat serum for 12 h at 4 °C. Following this, cells were stained with Cyanine5 (Cy5) conjugated goat anti-rabbit secondary antibody (Jackson ImmunoResearch, West Grove, PA; 1:100 dilution) for 1 h at room temperature. Post antibody staining, cell nuclei were stained with 4′,6-diamidino-2-phenylindole. The scaffolds were washed twice with 1× PBS at each step of the staining protocol and imaged at 20× using a confocal microscope (Nikon, Japan). Antigen specificity was confirmed by employing a negative control that entailed the same staining protocol but without the primary antibody.

### 2.12. Statistical analyses

Results are expressed as mean ± standard deviation. Data were analyzed using one-way or two-way ANOVA with Tukey post-hoc test for pairwise comparison (GraphPad, Prism, Boston, MA). Statistical analyses for comparison of print fidelity data with the STL generated model was performed using the one-sample *t*-test. Statistical significance criterion was set at *p* < 0.05.

## 3. Results

### 3.1. Effect of Col:XG composition and SMP concentration on rheological properties

**Figure 1.**
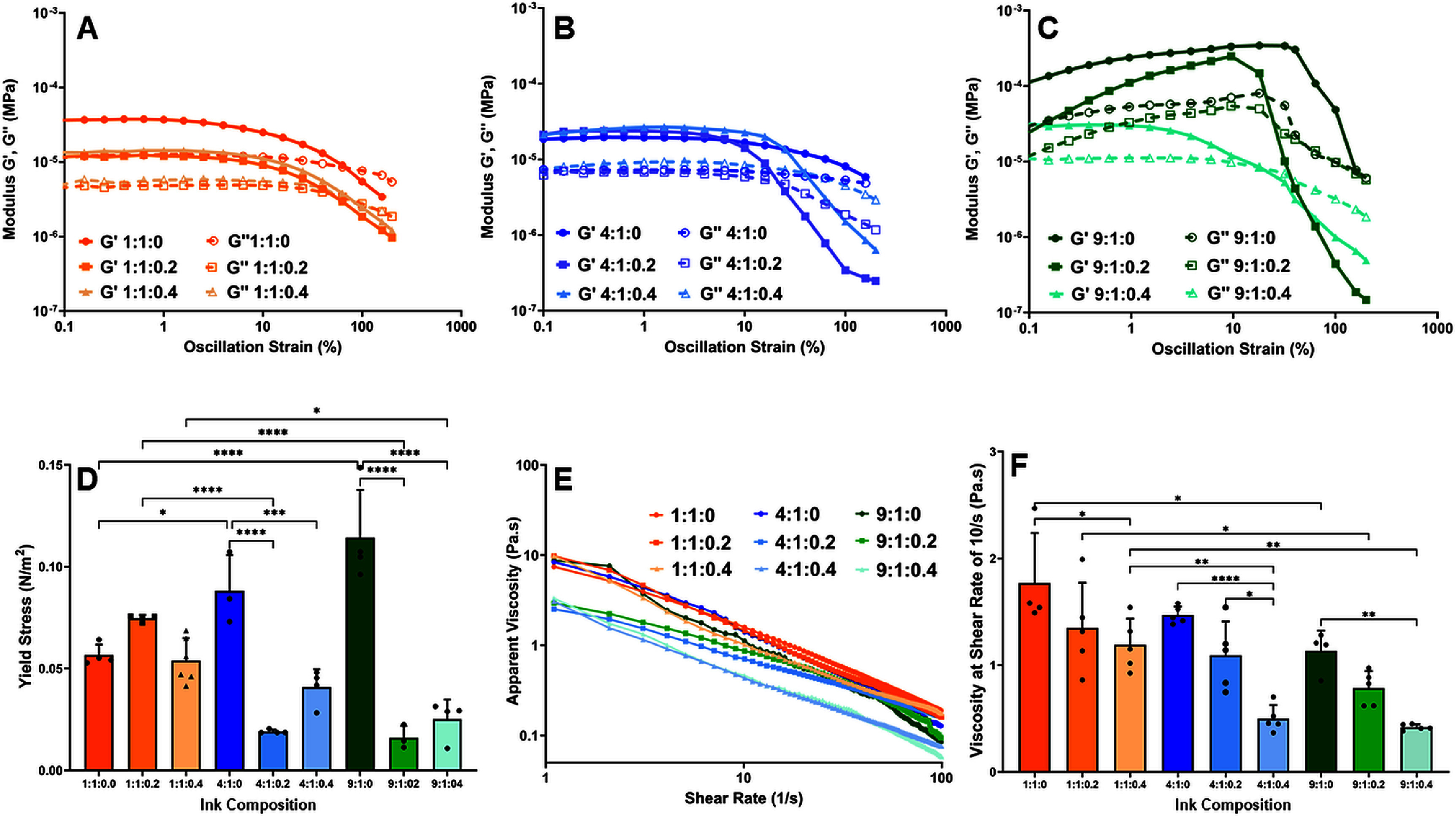
(A), (B), and (C) Storage (*G*′) and loss modulus (*G*″) vs. oscillation strain of printing inks depicted as Col:XG:SMP (D) yield stress of different printing inks (E) logarithmic plot of apparent viscosity vs. shear rate (F) viscosity of inks at a constant shear rate of 10 s^−1^. (* indicates *p* < 0.05, ** indicates *p* < 0.01, *** indicates *p* < 0.001, and **** indicates *p* < 0.0001).

The flow behavior of precursor inks is a crucial parameter to generate 3D scaffolds with high print fidelity. Results from the oscillation strain sweeps indicate that all inks exhibit viscoelastic behavior within a sweep region of 0.1%–10% strain indicated by the parallel curves of storage and loss modulus (figures 1(A)–(C)). Beyond this point the inks lose their viscoelastic properties (10%–100% strain region). The YS is determined at the point of crossover of the storage and loss modulus curves, beyond which the inks exhibit no viscoelastic behavior. SMP incorporation significantly decreased (*p* < 0.001) the YS of precursor inks with a Col:XG ratio of 4:1 and 9:1 (figure 1(D)). A similar decrease in YS upon SMP incorporation was not observed for inks with a Col:XG of 1:1 indicating that higher XG amount may mask the differences in the viscoelastic properties of inks caused by changes in SMP concentration (figure 1(D)).

The change in viscosity with respect to shear rate (flow ramp test) was used to determine the rheological properties of the precursor inks. Results demonstrate that all inks exhibit shear thinning properties as shown by the negative slope of the curves (figure 1(E)). Apparent viscosity, calculated at a constant shear rate of 10 s^−1^, was significantly lower (*p* < 0.05) for Col:XG inks with higher SMP concentration compared to no SMP (figure 1(F)). Additionally, at SMP concentration of 0.4 mg ml^−1^, the apparent viscosity of inks with a Col:XG composition of 1:1 was significantly higher (*p* < 0.01) than 4:1 and 9:1 Col:XG compositions. Overall, these results indicate that both Col:XG composition and SMP incorporation impact the rheological properties of the precursor inks.

### 3.2. Effect of Col:XG composition and SMP concentration on print fidelity

**Figure 2.**
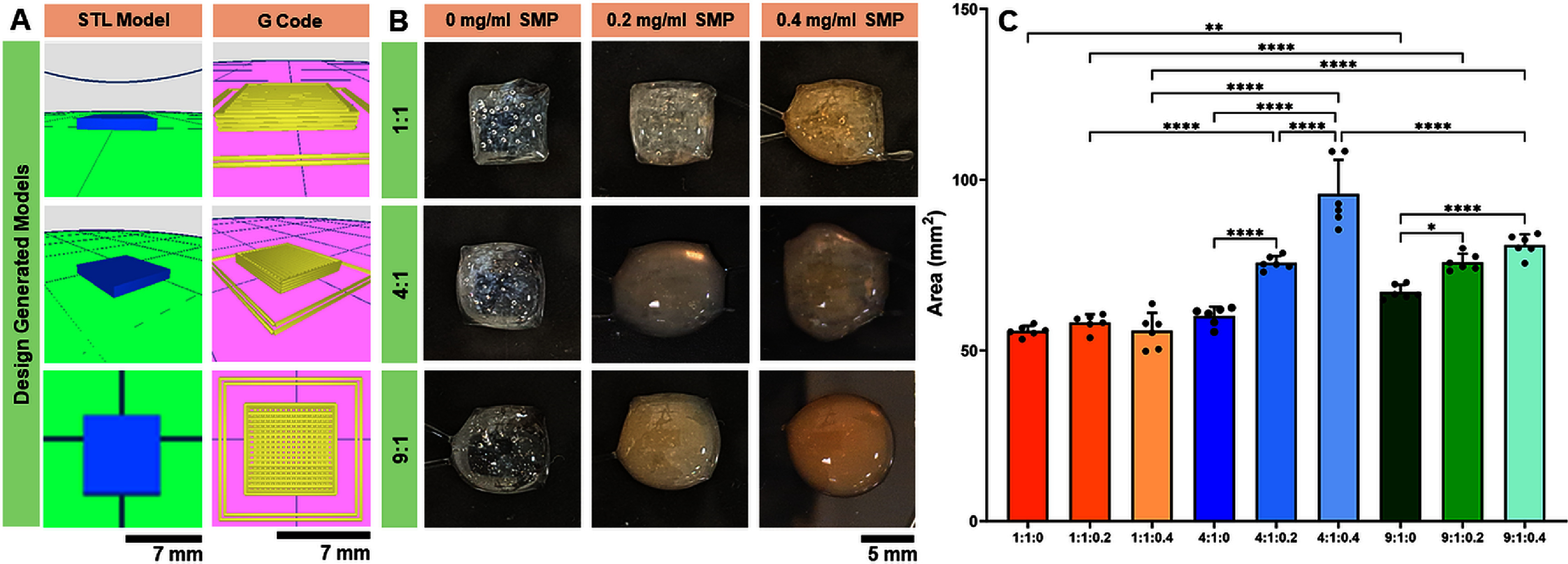
(A) Design generated STL model and rendered G-code for layer-by-layer printing. (B) Printability of inks composed of different Col:XG composition and SMP concentration. (C) Print fidelity measured by transverse sectional area (TSA) of the printed cuboid construct. (* indicates *p* < 0.05, ** indicates *p* < 0.01, *** indicates *p* < 0.001, and **** indicates *p* < 0.0001).

The effects of Col:XG composition and SMP incorporation on the printability of precursor inks was evaluated by comparing the TSA of 4D printed scaffolds using ImageJ. Visual examination of scaffolds post printing revealed that inks with a Col:XG ratio of 1:1 and no SMP showed the highest print fidelity (figure 2(A, B)). Print fidelity was adversely impacted by increase in SMP concentration and decrease in XG composition. Quantitative assessment of the areas of the printed scaffolds indicated that SMP incorporation had no effect on print fidelity when using Col:XG ink with 1:1 composition (figure 2(C); TSA ∼ 55 mm^2^). However, for 4:1 and 9:1 Col:XG compositions, increase in SMP concentration resulted in significantly higher (*p* < 0.01) area measurements (i.e. 75–90 mm^2^) indicating decrease in print fidelity. For a fixed SMP concentration, use of higher XG composition (Col:XG 1:1) yielded printed scaffolds with significantly greater print fidelity (*p* < 0.001) when compared to Col:XG compositions of 4:1 and 9:1. Together, results for print fidelity are in agreement with the rheological findings indicating that changes in ink viscosity triggered by modulating Col:XG composition and SMP concentration impact the fidelity of the printed scaffolds.

### 3.3. Effect of Col:XG composition and SMP concentration on collagen fiber alignment

**Figure 3.**
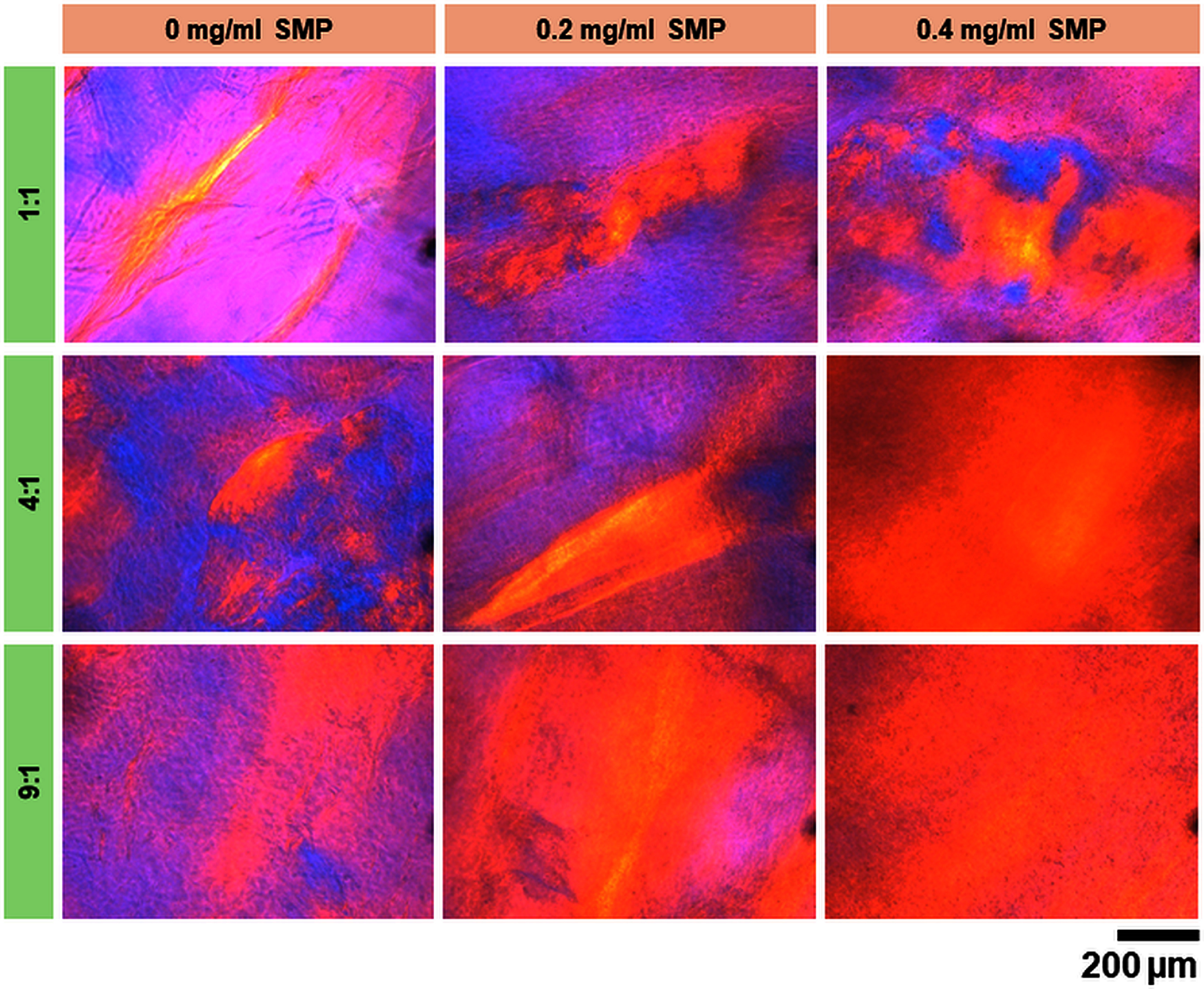
PLM images showing presence of collagen alignment indicated by even bright yellow color in 4:1 and 9:1 Col:XG scaffolds with 0.4 mg ml^−1^ SMP concentration.

Collagen fiber alignment in 4D printed scaffolds was evaluated using PLM and SEM. Results from PLM imaging showed a prominent bright yellow hue in scaffolds printed using 4:1 Col:XG ink incorporated with 0.4 mg ml^−1^ SMP indicating the presence of collagen anisotropy uniformly throughout the construct (figure 3). At the same SMP concentration, Col:XG scaffolds printed using 9:1 Col:XG showed a similar bright yellow color with lesser uniformity and lower intensity of the yellow hue, while scaffolds printed using 1:1 Col:XG composition showed minimal yellow color in an irregular and non-uniform pattern. Use of SMP at a lower concentration (0.2 mg ml^−1^) yielded scaffolds with partial collagen alignment as evidenced by patchy appearance in the yellow hue. Scaffolds printed with no SMP showed bright pink and blue hues with sparse streaks of bright yellow indicating little to no collagen fiber alignment.

**Figure 4.**
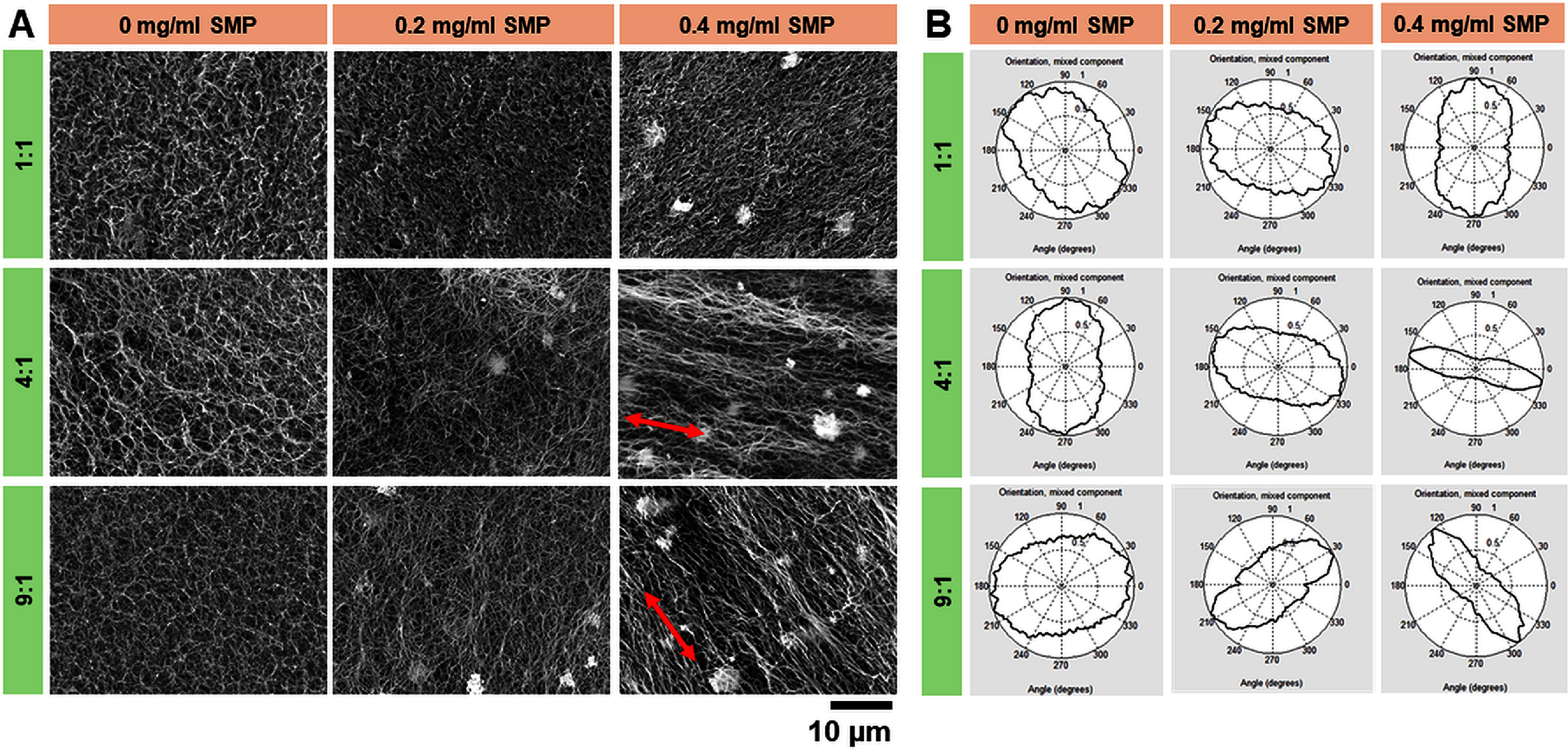
(A) SEM images for evaluation of collagen fiber alignment. Red arrows indicate direction of collagen fiber orientation. (B) SEM quantification showing distribution of angular orientation of collagen fibers depicted as a 360° plot.

SEM imaging revealed that scaffolds printed using Col:XG inks at a ratio of 1:1 composed of compact and denser collagen fiber packing, while the all other scaffolds displayed a classic collagen fibrillar structure (figure 4(A)). The results from SEM imaging corroborated with the PLM findings and showed that the highest degree of collagen fiber alignment was observed in scaffolds printed using 4:1 and 9:1 Col:XG ink composition and 0.4 mg ml^−1^ SMP (figure 4(A)). Scaffolds printed using 0 or 0.2 mg ml^−1^ SMP showed no evidence of collagen fiber alignment for any of the Col:XG ink compositions (figure 4(A)). These findings were further confirmed by quantification of collagen fiber alignment in SEM images via FFT analysis. Quantitative results showed that the angular distribution plots for scaffolds printed using 4:1 or 9:1 Col:XG composition and 0.4 mg ml^−1^ SMP exhibited the narrowest spread in fiber orientation angle, while a greater spread in angular distribution was observed for no SMP and 0.2 mg ml^−1^ SMP concentrations, as well as for 1:1 Col:XG composition for all SMP concentrations (figure 4(B)). Together, the results from PLM and SEM imaging suggest that Col:XG composition of 4:1 and SMP concentration of 0.4 mg ml^−1^ are optimal conditions for producing 4D printed scaffolds with high degree of collagen fiber alignment.

### 3.4. Modulation of print flow speed to improve the fidelity of 4D printed collagen scaffolds

**Figure 5.**
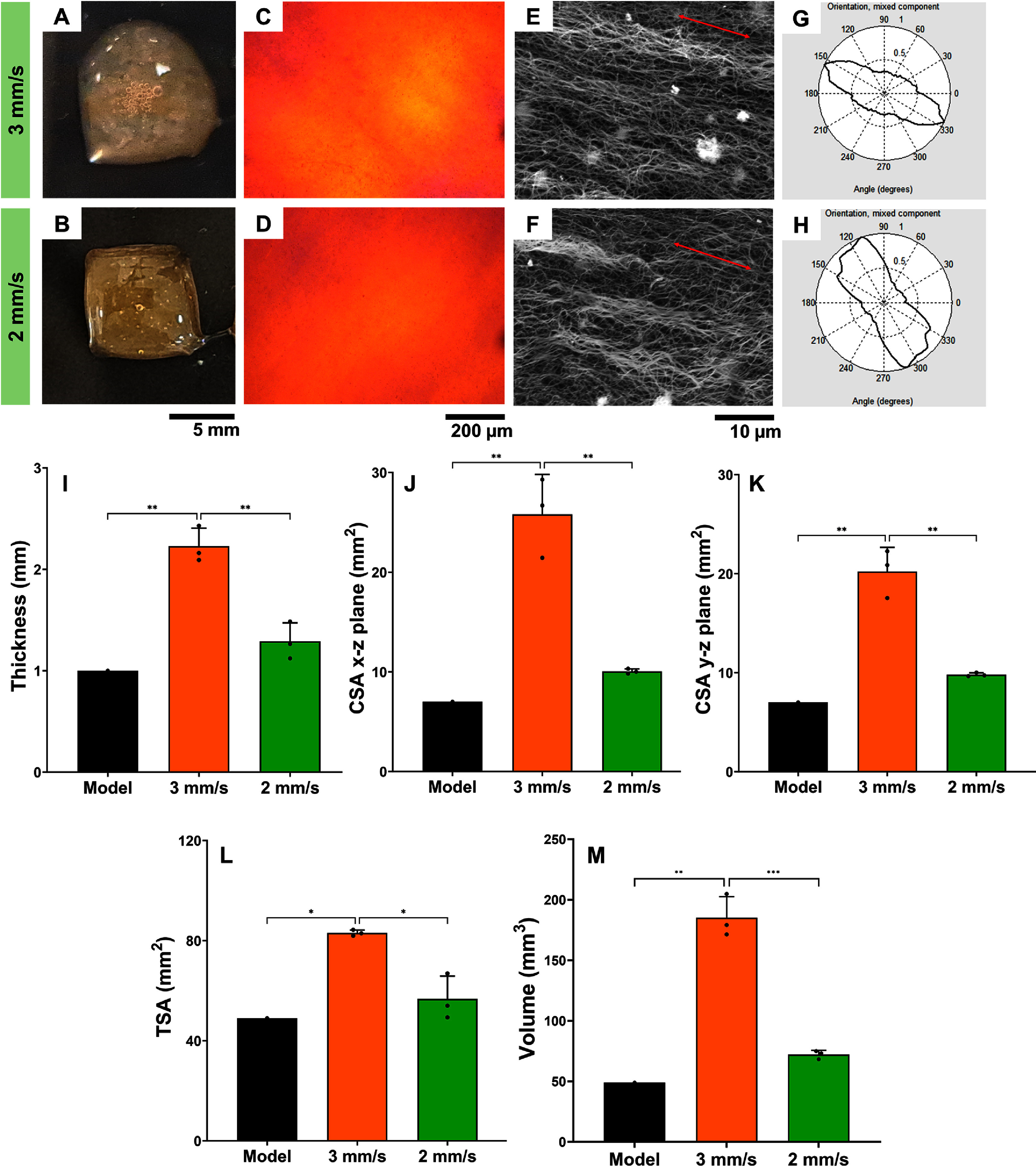
Printability of magnetically aligned construct using a print speed of (A) 3 mm s^−1^ (B) 2 mm s^−1^. (C), (D) Confirmation of collagen fiber alignment using PLM. (E), (F) Confirmation of collagen fiber alignment using SEM. Red arrows indicate direction of fiber orientation. (G), (H) SEM quantification showing distribution of angular orientation of collagen fibers depicted as a 360° plot. (I) Scaffold thickness measured by confocal microscopy. (J) Print area fidelity measured by cross-sectional area (CSA) of the printed cuboid scaffold in the *x*–*z* plane. (K) Print area fidelity measured by cross-sectional area (CSA) of the printed cuboid scaffold in the *y*–*z* plane. (L) Print area fidelity measured by transverse sectional area (TSA) of the printed cuboid scaffold. (M) Print volume fidelity measured by multiplying the thickness with the TSA of the printed scaffold (* indicates *p* < 0.05, ** indicates *p* < 0.01, and *** indicates *p* < 0.001).

While scaffolds printed using 4:1 Col:XG and 0.4 mg ml^−1^ SMP showed the highest degree of collagen fiber alignment, the print fidelity of these scaffolds was significantly poor when compared to the initial generated STL model (figure 5). To improve the printability, the flow speed was modified to 2 mm s^−1^ instead of 3 mm s^−1^ used in prior experiments. Results showed that print area fidelity improved when flow speed of 2 mm s^−1^ was used as indicated by the TSA of the printed scaffold which was significantly lower compared to that of scaffolds printed using a flow speed of 3 mm s^−1^ (figures 5(A) and (B)). PLM and SEM imaging confirmed that collagen fiber alignment was retained for both flow speed conditions (figures 5(C)–(H)). Quantitative analyses revealed that the scaffolds printed using a flow speed of 3 mm s^−1^ showed significantly higher (*p* < 0.05) measurements (thickness ∼ 2.2 mm, CSA (*x*–*z* plane) ∼ 25 mm^2^; CSA (*y*–*z* plane) ∼ 20 mm^2^; TSA ∼ 80 mm^2^, *p* < 0.05) compared to the STL model (thickness = 1 mm, TSA = 49 mm^2^, CSA = 7 mm^2^) indicating poor print fidelity (figures 5(I)–(L)). On the other hand, use of a lower flow speed of 2 mm s^−1^ yielded scaffolds with measurements that were significantly lower compared to scaffolds printed using a flow speed of 3 mm s^−1^ (thickness ∼ 1.3 mm, CSA (*x*–*z* plane) ∼ 10 mm^2^; CSA (*y*–*z* plane) ∼ 10 mm^2^; TSA ∼ 55 mm^2^, *p* < 0.05) and were comparable to that of the STL model (*p* = 0.24 for thickness, figure 5(I); *p* = 0.65 for CSA (*x*–*z* plane), figure 5(J); *p* = 0.42 for CSA (*x*–*z* plane), figure 5(K); *p* = 0.36 for TSA, figure 5(L)). Volume measurements revealed that scaffolds printed using a print flow speed of 3 mm s^−1^ showed a significantly poorer volume fidelity (185 mm^3^; *p* < 0.01) compared to the volume of the designed STL model (49 mm^3^). In contrast, the scaffolds printed using a 2 mm s^−1^ flow speed retained the print volume fidelity by showing a volume (72 mm^3^) comparable to that of the generated STL model (*p* = 0.35; figure 5(M)). Based on these findings, employing a slower print flow speed of 2 mm s^−1^ resulted in better retention of shape fidelity in thickness, area, and volume of the printed scaffold. The optimal conditions of 4:1 Col:XG composition, 0.4 mg ml^−1^ SMP, and 2 mm s^−1^ print flow speed were used for the ensuing cell culture studies.

### 3.5. Impact of collagen fiber alignment on hMSC morphology and cell metabolic activity

**Figure 6.**
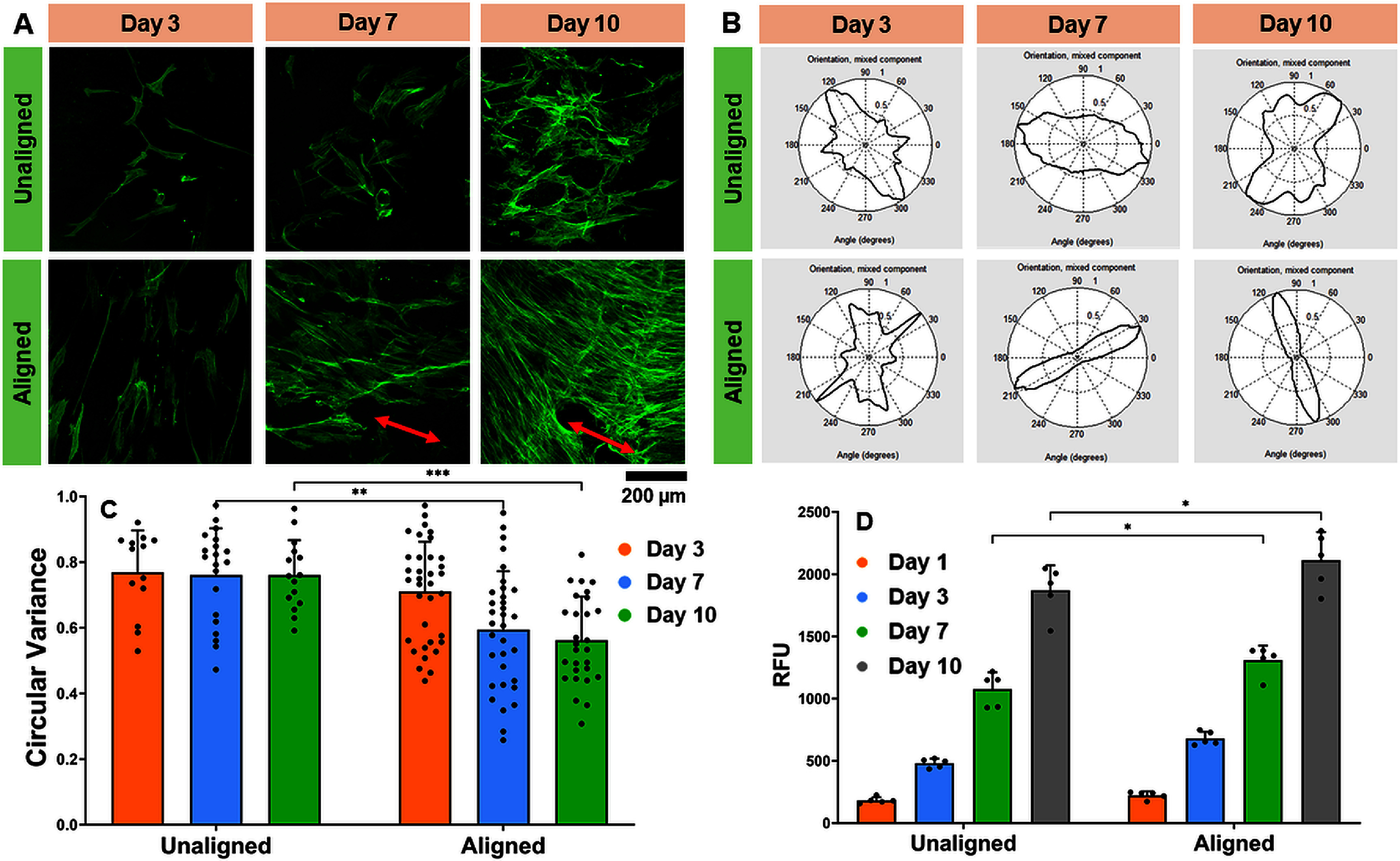
(A) Assessment of cell morphology via cytoskeleton staining using Alexafluor Phalloidin 488. Red arrows indicate direction of cellular orientation. (B) Quantification of cell orientation distribution depicted as a 360° plot. (C) Circular variance between 0 and 1, where 0 indicates perfect anisotropy and 1 indicates no preferred direction of cell orientation. (D) AlamarBlue assay showing cell metabolic activity. (* indicates *p* < 0.05, ** indicates *p* < 0.01, and *** indicates *p* < 0.001).

The effect of collagen fiber alignment on the morphology of hMSCs was evaluated using cell cytoskeleton staining. Results showed no preferential direction of cellular alignment on scaffolds printed in the absence of magnetic field (i.e. unaligned), while cells cultured on scaffolds printed with the magnetic field (i.e. aligned) showed preferential orientation at day 7 and day 10 (figure 6(A)). Quantification of cellular alignment using FFT analysis showed a narrow angular distribution for cells cultured on aligned scaffolds compared to unaligned scaffolds (figure 6(B)). These results were corroborated by the circular variance measurements which showed a value of around 0.5 for cellular orientation on aligned scaffolds at day 7 and day 10 which was significantly lower than the circular variance of cellular orientation measured on unaligned scaffolds (figure 6(C)). Alamar blue assay results showed an increase in RFU with time for both unaligned and aligned collagen scaffolds. Further, significantly higher (*p* < 0.05) RFU was observed on aligned collagen scaffolds at day 7 and day 10 indicating that collagen anisotropy either promotes greater cell proliferation, induces higher metabolic activity per cell, or both (figure 6(D)). More importantly, these results suggest that cell viability is maintained on both unaligned and aligned collagen scaffolds indicating that the components added to the collagen ink (i.e. VA-086, XG, SMP) are not cytotoxic.

### 3.6. Impact of collagen fiber alignment on expression of SCXA via immunofluorescence

**Figure 7.**
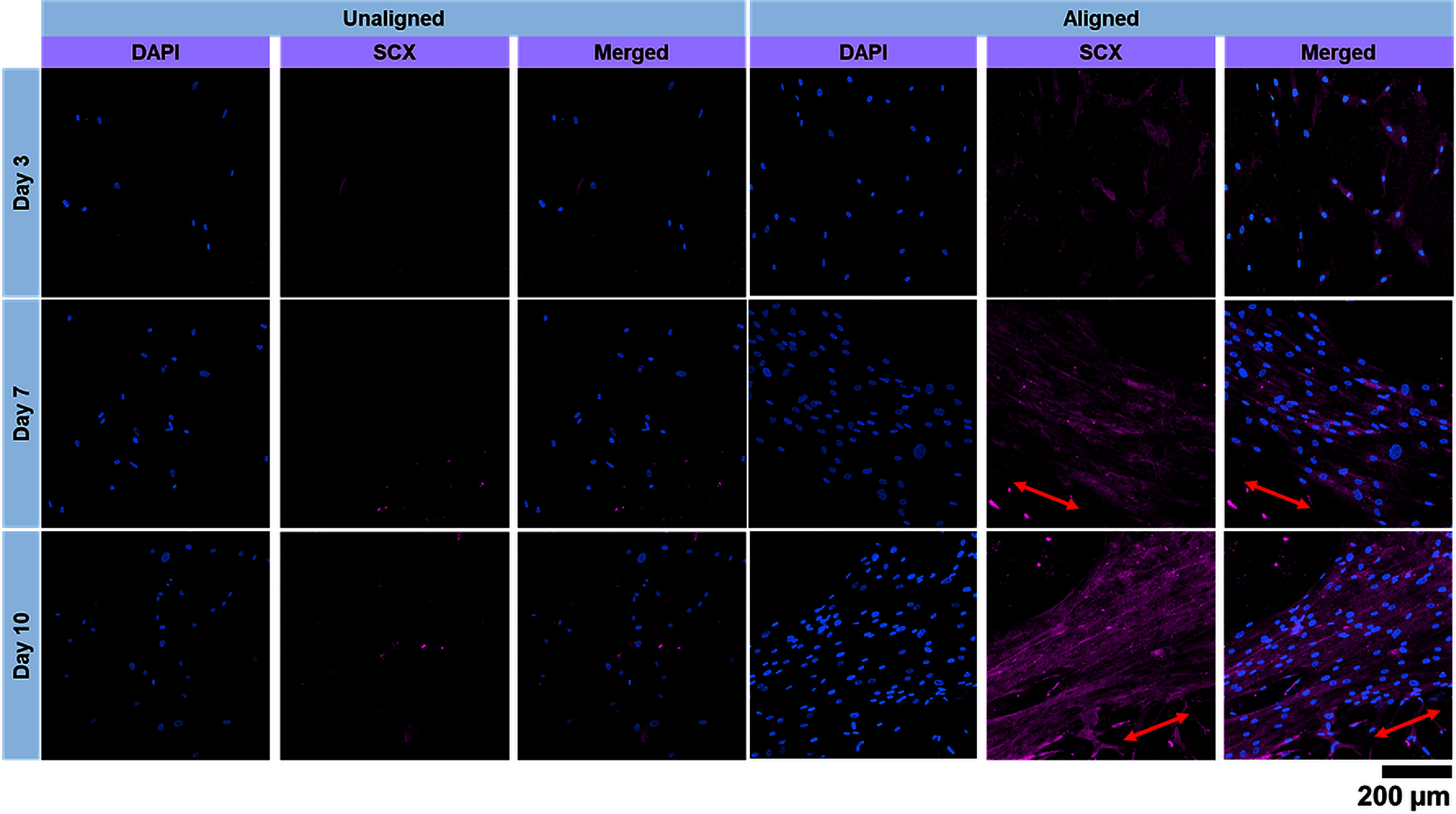
Immunofluorescence staining for expression of scleraxis (SCXA) on unaligned and aligned collagen scaffolds. Red arrows indicate the direction of cellular orientation.

Immunofluorescence staining results showed little to no expression of SCXA on unaligned collagen scaffolds at all time points (figure 7). On the contrary, clear evidence of SCXA expression localized around the cell nuclei was observed very early in culture (i.e. day 3) on aligned collagen scaffolds. Further, SCXA expression was found to increase on aligned collagen scaffolds as the cell number increases with the duration of the culture. In addition, visual assessment of the images revealed a possible increase in SCXA expression on aligned collagen scaffolds over time as indicated by the higher staining intensity when comparing day 7 and day 10 to day 3. Together, these results clearly suggest that collagen anisotropy triggers ligament-like differentiation of hMSCs.

## 4. Discussion

This work presents a novel 4D printing strategy that combines extrusion-based 3D printing and a magnetic alignment approach to generate reproducible multilayered scaffolds with a high degree of collagen fiber alignment. The relevance lies in incorporating a fourth dimension to leverage real-time matrix remodeling of fiber-forming collagen molecules, to generate highly anisotropic scaffolds that recapitulate the native microarchitecture of ligamentous tissue. The progression of this study included (1) modulation of Col:XG ink composition to generate reproducible collagen scaffolds with high print fidelity by assessing the rheological properties of the inks and performing printability studies; (2) optimization of SMP concentration and printing parameters to produce highly aligned collagen scaffolds, and (3) assessment of hMSC behavior on aligned collagen scaffolds to demonstrate applicability for musculoskeletal tissue engineering.

Collagen inks have low viscosity and hence extrusion-based 3D printing is typically performed using a support bath that retains the fidelity of the printed scaffold. For example, prior work using freeform embedded suspended hydrogels (FRESHs) as a support bath has shown that it is feasible to print complex multilayered collagen scaffolds with good print fidelity [24, 25]. One possible limitation of the FRESH technique is challenges with reproducibility due to uneven melting of the support bath that generates shear forces triggering scaffold disintegration [26]. In addition, printing in a highly viscous support bath will hinder movement of SMPs in response to the magnetic field which is the driving factor for collagen alignment in the printed scaffold. Recent work has demonstrated that it is possible to print collagen scaffolds in air (i.e. w/o support bath), by combining biocompatible additives (e.g. XG) with collagen to increase the viscosity of the ink solution [27, 28]. Results of this study showed that collagen inks exhibited higher viscosity with the addition of XG at 1:1 Col:XG composition for all SMP concentrations and yielded scaffolds with good print fidelity (figures 1 and 2). Decrease in YS and viscosity of collagen inks with lower XG composition (4:1 and 9:1 Col:XG) and SMP incorporation adversely impacted the print fidelity of scaffolds. Shape retention of the printed scaffolds depend on the underlying material properties such as viscoelasticity and YS that greatly impact the recovery kinetics, ink resistance to deformation, and vertical printability of multilayered scaffolds [29, 30]. In addition, results of this study demonstrated that printing parameters such as print flow speed also greatly impact the shape retention of the scaffold indicated by scaffolds thickness, CSA, TSA, and volume measurements which were comparable to that of the generated STL model when a print speed of 2 mm s^−1^ was used, while a higher print speed (3 mm s^−1^) resulted in greater ink deposition and yielded scaffolds with poorer shape retention and print fidelity (figure 5). CSA measurements revealed that shape retention of the printed scaffold was consistent between the two planes (i.e. *x*–*z* and *y*–*z*), especially when using a print flow speed of 2 mm s^−1^ (figures 5(J) and (K)).

Magnetic alignment can be achieved by exploiting the diamagnetic properties of collagen to trigger orientation of the collagen molecules in a direction perpendicular to the applied magnetic field. While this process generates uniform and high degree of collagen fiber alignment, the low diamagnetism of collagen mandates a magnetic field strength in the range of 6–12 Tesla that typically require very strong electromagnets [31, 32]. To reduce the risk of equipment-related complications as well as the associated costs of electromagnets, application of magnetic particle-guided collagen alignment with low-level magnetic fields (<0.5 Tesla) has emerged as a promising strategy to generate aligned collagen scaffolds [33–35]. Upon exposure to low-level magnetic fields, the magnetic particles orient parallel to the direction of the field and simultaneously guide the alignment of small collagen fibers parallel to the direction of particle orientation. These fibers then act as nucleation sites for long-range collagen fiber formation and elongation during the fibrillogenesis process in the desired direction of orientation [36]. In this context, use of inks with 1:1 Col:XG composition yielded scaffolds with poor collagen fiber alignment possibly due to hindrance of magnetic particle movement in inks with higher viscosity (figures 3 and 4). On the contrary, 0.4 mg ml^−1^ SMP incorporation in 4:1 and 9:l Col:XG compositions decreased the ink viscosity and hence showed the highest degree of collagen fiber alignment but suboptimal print fidelity. Modulation of print flow speed enabled retention of collagen fiber alignment and improvement in print fidelity of scaffolds printed using 4:1 Col:XG composition and 0.4 mg ml^−1^ SMP concentration (figure 5). Reduction in print flow speed decreases the shear stress imparted on the ink and helps better retain the viscoelastic properties to enable printing of collagen scaffolds with higher fidelity. Prior studies that employ magnetic-bead driven collagen alignment have reported short-range, non-uniform, and partial degree of collagen fiber orientation [36–38]. Optimization of the ink composition and print parameters in the current study delivered scaffolds with more uniform collagen fiber alignment over a larger area of spatial distribution.

Cell studies were performed on collagen scaffolds fabricated using the optimal conditions identified in this work (i.e. 4:1 Col:XG, 0.4 mg ml^−1^ SMP, 2 mm s^−1^ print flow speed) with (aligned) and without (unaligned) the presence of the magnetic field. Results showed that cell metabolic activity increased over time on both aligned and unaligned scaffolds indicating that XG and SMP have no cytotoxic effects (figure 6). In addition, clear evidence of cell orientation was observed on aligned collagen scaffolds which may be mediated by the oriented SMPs as well as the aligned collagen fibers (figure 6). Prior studies have shown that topographical cues from aligned collagen scaffolds can guide tenogenic differentiation of human MSCs without the addition of any external factors [8, 38, 39]. In this work, immunofluorescence assay was performed with SCXA, a well-established early marker of tenogenic and ligamentous differentiation, to demonstrate the applicability of 4D printed collagen scaffolds in repair and regeneration of soft tissues such as tendons and ligaments. Results showed that SCXA was expressed very early in culture (day 3) and continued to increase with time on aligned collagen scaffolds indicating that hMSCs can sense and respond to the topographical cues provided by collagen anisotropy and differentiate specifically to the ligamentous lineage (figure 7). These results agree with prior studies that reveal that aligned collagen topography is a key driving factor for tenogenic/ligamentous differentiation of MSCs [40, 41]. One limitation of the current study is that the culture duration was constrained to 10 d due to cell-mediated contraction of the collagen scaffold. Longer duration cultures will require crosslinking of the scaffolds using reagents such as genipin to improve the mechanical properties and thereby resist scaffold contraction post cell confluency. While the mechanical properties of 4D printed scaffolds reported in this work are weak, optimal genipin crosslinking can improve the mechanical properties of the scaffold comparable to that of the native tissue and thereby enhancing applicability towards repair and regeneration of load-bearing tissues [42].

In conclusion, the results from this study suggest that optimization of ink composition and print parameters can deliver 4D printed scaffolds with good shape fidelity and high degree of collagen fiber alignment. Introduction of collagen anisotropy in 4D printed scaffolds guides cell orientation and triggers ligamentous differentiation of hMSCs. Overall, 4D printing is a promising strategy to generate multilayered anisotropic collagen scaffolds for use in ligament and tendon tissue engineering applications.

## Data Availability

All data that support the findings of this study are included within the article (and any supplementary files).
